# Association Between Advanced Care Management and Progression of Care Needs Level in Long-Term Care Recipients: Retrospective Cohort Study

**DOI:** 10.2196/11117

**Published:** 2018-07-25

**Authors:** Sakiko Itoh, Hiroyuki Hikichi, Hiroshi Murayama, Miho Ishimaru, Yasuko Ogata, Hideo Yasunaga

**Affiliations:** ^1^ Nursing Career Pathway Center Department of Gerontological Nursing and Care System Development, Graduate School of Health Care Sciences Tokyo Medical and Dental University Tokyo Japan; ^2^ Department of Social and Behavioral Sciences Harvard TH Chan School of Public Health Boston, MA United States; ^3^ Institute of Gerontology The University of Tokyo Tokyo Japan; ^4^ Department of Clinical Epidemiology & Health Economics School of Public Health The University of Tokyo Tokyo Japan

**Keywords:** community health services, health services for the aged, integrated care, long-term care, patient care planning

## Abstract

**Background:**

Long-term care insurance systems in Japan started a special senior care program overseen by qualified care managers (also known as advanced care managers). However, the relationship between advanced care management and outcomes in long-term care recipients remains unknown.

**Objective:**

We aimed to compare the outcome of long-term care recipients using facilities with advanced care management and conventional care management, in terms of care needs level progression.

**Methods:**

We conducted a retrospective cohort study using the Survey of Long-Term Care Benefit Expenditures in Japan. We identified those aged ≥65 years who were newly designated a care need level of 3, and received long-term care services between April 2009 and March 2014 in Tokyo. We compared survival without progression of care needs level between the groups, with and without advanced care management, using the Kaplan-Meier method. Factors affecting the outcomes were determined using a multivariable logistic regression model fitted with a generalized estimating equation.

**Results:**

Of 45,330 eligible persons, 12,903 (28.46%) received long-term care based on advanced care management. The average duration of progression-free survival was 17.4 (SD 10.2) months. The proportions of five-year cumulative progression-free survival were 41.2% and 32.8% in those with and without advanced care management, respectively. The group with advanced care management had significantly lower care needs levels (odds ratio 0.77, 95% CI, 0.72-0.82, *P*<.001).

**Conclusions:**

Advanced care management was significantly associated with improved care needs levels.

## Introduction

In 2000, the Japanese government introduced Long-Term Care Insurance (LTCI) for the elderly, which provides welfare and health care services with comprehensive care management [1-3]. Eligible persons can receive a variety of services, such as nurses’ visit, rehabilitation at home, bathing service at home, meal services at home, and rental service for welfare equipment [4-6]. Long-term care support specialists called “care managers” oversee the services provided by nurses, physical therapists, and professional caregivers [7-9]. The main tasks of care managers are: (1) assessing care needs and health problems of the elderly and their families; (2) coordinating care providers and designing long-term care service plans and care programs; and (3) monitoring and evaluating the long-term care service plans and care programs [10-11].

In 2006, the government introduced the higher-level position of “advanced care manager,” who must have at least five years of experience as a care manager and are expected to design higher quality long-term care service plans and care programs [4,12]. In April 2009, the government began additional payments (also known as "care management premiums") for long-term care agencies with advanced care managers [13]. However, little is known about the effectiveness of this governmental policy and whether it has improved long-term care in Japan. Specifically, it remains unclear whether care coordination by advanced care managers is associated with improved outcomes of elderly persons. In the present study, we compared the progression of care needs levels of long-term care recipients with advanced care management and conventional care management, using a national long-term care database in Japan.

## Methods

### Study Design and Data Source

The study design was a retrospective cohort study. We used data from Japan’s Ministry of Health, Labour and Welfare that was collected as part of the Survey of Long-Term Care Benefit Expenditures [14]. This national data set contained baseline characteristics and information on living arrangement, dementia, care needs levels (ranging from 1 to 5), type of long-term care services, type of long-term care agencies, and care managers in charge of care plans.

The targeted population was elderly persons, who were eligible for the LTCI services in Tokyo between April 2009 and March 2014. The study subjects were selected based on the following criteria: (1) persons of ≥65 years; (2) those who were newly designated a care needs level of 3; and (3) using long-term care service plans and care programs that were designed by care managers. Care needs level 3 indicates a moderate level of assistance required for everyday activities, such as standing, excretion, and bathing. We excluded individuals who were: (1) using in-home services for less than six months; and (2) not using in-home services for more than one month.

### Measurements

#### The Outcome

The outcome of the present study was progression in care needs level. This was measured according to the criteria of “independence degree of daily living for the elderly,” which was created by the government to assign elderly persons a score from 1 (less dependent) to 5 (more dependent) [1,4,6,15]. Care needs levels were normally reassessed every 12 months.

#### Advanced Care Management

The advanced care manager system was introduced to improve quality of care management for long-term care recipients [4,12,16]. Advanced care managers must have more than five years of experience as a care manager [17,18]. The government started care management premiums for long-term care agencies with at least one advanced care manager under the LTCI in 2009 [13].

### Data Analysis

#### Progression-Free Survival Analysis

We used the Kaplan-Meier method and log-rank tests to compare survival without progression of care needs levels (ie, transition from care level 3 to 4 or 5) between the groups with and without advanced care management. The progression-free period was counted from the month participants received their care needs level 3 designation to the month care levels started to change, indicating a decline in health. Censoring criteria were: (1) no events, (2) hospital admission or nursing home admission, and (3) death during the study period.

#### Risk Factors

To examine risk factors associated with progression in care needs level, we used a logistic regression model fitted with a generalized estimating equation to account for the differences in follow-up period lengths. The dependent variable was the progression of care needs levels. The predictor variables included: (1) demographic variables (age, gender, and living alone); (2) dementia (level of independent living ≥3); and (3) type of agency with and without advanced care management. These variables were based on a previous study and existing knowledge of risk factors for the progression of care need levels [19]. The threshold for statistical analyses was set at *P*<.05 in a two-tailed test. Statistical analyses were performed using Stata (version 14, Stata Corp, Texas, USA).

### Ethical Considerations

Ethical considerations were examined in accordance with the Japanese epidemiological guidelines for secondary data analysis [20]. This study was approved by the Institutional Review Board at The University of Tokyo, Japan. The requirement for informed consent was waived because of the anonymized nature of the data in the database.

## Results

We identified 45,330 eligible people during the study period. The baseline information is shown in Table 1. The number of elderly people with and without advanced care management were 12,903 (28.46%) and 32,427 (71.53%), respectively. The progression-free period lasted on average for 17.4 (SD 10.2) months. A total of 10,327 patients had an increased care needs level.

**Figure 1:**
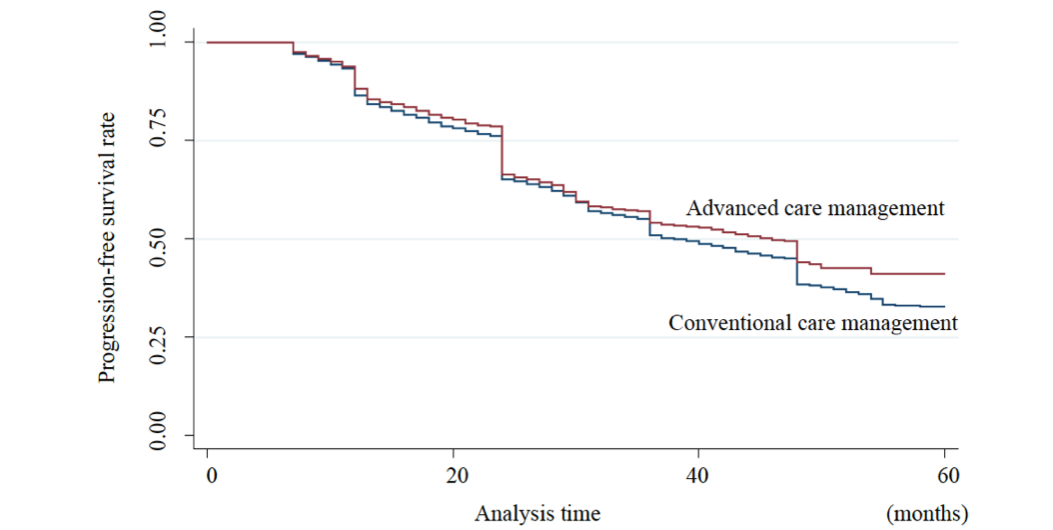
The Kaplan-Meier method for care level progression. Analyzing care level change in 45,330 patients showed that the 5-year cumulative progression-free survival rate of the special and the general agencies were 41.2% and 32.8%, respectively.

Figure 1 shows the progression-free survival curves in the groups with and without advanced care management. The proportions of five-year cumulative progression-free survival in the groups with and without advanced care management were 41.2% and 32.8%, respectively (*P*<.001). The hazard ratios of the groups were not constant over the study period, having changed at the regular reassessment months (eg, 6, 12, 24, 36, and 48 months).

Table 2 summarizes the risk factor analysis indicating that those with advanced care management were significantly less likely to experience increasing levels of care (odds ratio [OR] 0.77, 95% CI 0.72-0.82, *P*<.001). Age was significantly associated with progression of care needs level (OR 1.01, 95% CI 1.01-1.02, *P*<.001).

**Table 1:** Study population treated in long-term care agency in 2009-2014. A total of 29,815,241 receipt data were analyzed in 45,330 patients during the analysis for 5 years.

Variable			Value	
**Elder person characteristics**				
	Age (years), mean (SD)			82.7 (7.83)
	**Gender, n (%)**			
		Male		17,113 (37.75)
		Female		28,217 (62.25)
	**Living alone, n (%)**			
		Yes		6465 (14.26)
		No		38,865 (85.74)
	**Dementia, n (%)**			
		Level of independent living ≥3		11,262 (24.84)
		Other		34,068 (75.16)
**Agency characteristics, n (%)**				
	**Types of management**			
		Advanced care management		12,903 (28.46)
		Conventional care management		32,427 (71.54)

**Table 2:** Risk factor analysis for care level progression using a generalized estimating equations model. The generalized estimating equations model showed that age and dementia were risk factors associated with care level progression. Advanced care management and living alone reduced the risk for deterioration of care levels.

Variable^a^	Odds ratio (95% CI)	*P* value
Age (years)	1.01 (1.01-1.02)	<.001
Gender (female)	1.04 (0.99-1.08)	.13
Living alone (yes)	0.81 (0.76-0.87)	<.001
Dementia (yes)	1.40 (1.33-1.47)	<.001
Type of management (advanced care management)	0.77 (0.72-0.82)	<.001

^a^All variables were entered into the statistical analysis (forced entry).

## Discussion

### Primary Findings

We found that elderly persons with advanced care management were significantly less likely to experience care needs level progression than those without it. Previous small studies suggested that advanced care managers could appropriately assess and care for elderly persons based on their extensive experiences [21,22]. They could combine a variety of care services efficiently and design them suitably due to their extensive knowledge of the subject. They were also more likely to coordinate care teams effectively with their vast experience in long-term care. Therefore, the advanced care managers can improve care service plans and programs, which may have a meaningful impact on the outcomes of older persons.

Furthermore, agencies with advanced care managers can provide better educational environments than those without, since one of their basic duties of these professionals is training and supporting other care managers [21,23]. We believe such an educational environment may have an impact on the outcomes discussed in this paper. Further studies are needed to assess this.

In our risk factor analysis, age and dementia were risk factors for the progression of care needs levels. These results were consistent with those in previous studies [24-26]. Studies conducted in other countries also identified age and dementia as major risk factors for the deterioration of functional levels amongst the elderly [27-29]. Regarding the hazard ratio in the progression-free survival analysis, we noted ratio changes in both groups at the regular reassessment months. It is possible that some older adults did not apply for the reassessments when their functional and psychosocial statuses deteriorated seriously. Additional research focusing on the reassessments is needed.

#### Policy Implications

The care management premiums for advanced care management was introduced to enhance health care service quality and improve outcomes [13]. Without any evidence for the efficacy of this policy, there has been on-going discussions about additional preferential treatment for long-term care agencies with advanced care managers [30]. Although our study did not analyze the direct effect of this policy, we believe additional preferential treatment for advanced care management may be useful for improving health care among the elderly.

#### Study Limitations

Our study has several limitations. First, because the present study was based on an observational design, there may have been unmeasured confounding factors that could have affected the progression of care needs levels. The national survey did not capture diagnosis, medications, family history, educational status, living status, or living areas in Tokyo. To investigate the effects of these factors, it would be important to reassess the data of individual recipients in the future. As for demographic characteristics, a previous report showed similar demographic characteristics across agencies [31]. Second, our findings may not be generalized to other countries.

### Conclusion

The present study used a national long-term care database to show that elderly persons utilizing advanced care management had a lower probability of deteriorating health (eg, progression of care needs levels). Our findings suggest that advanced care management may be effective for improving the outcome of long-term care recipients.

## Appendix

Multimedia Appendix 1 Flow diagram (45,330 eligible people identified during the study period).

Multimedia Appendix 2 Study population treated in long-term care agency in 2009-2014 by type of care management.

## Abbreviations

LTCI: Long-Term Care Insurance
OR: odds ratio
