# Supplementary figures and images for: Association Between Advanced Care Management and Progression of Care Needs Level in Long-Term Care Recipients: Retrospective Cohort Study
Source: JMIR Aging. 2018 Jul 25;1(2):e11117. doi: 10.2196/11117 (PMC6716439; doi:10.2196/11117)

## Supplemental Data 1

Follow Diagram. We identified 45,330 eligible people during the study period.

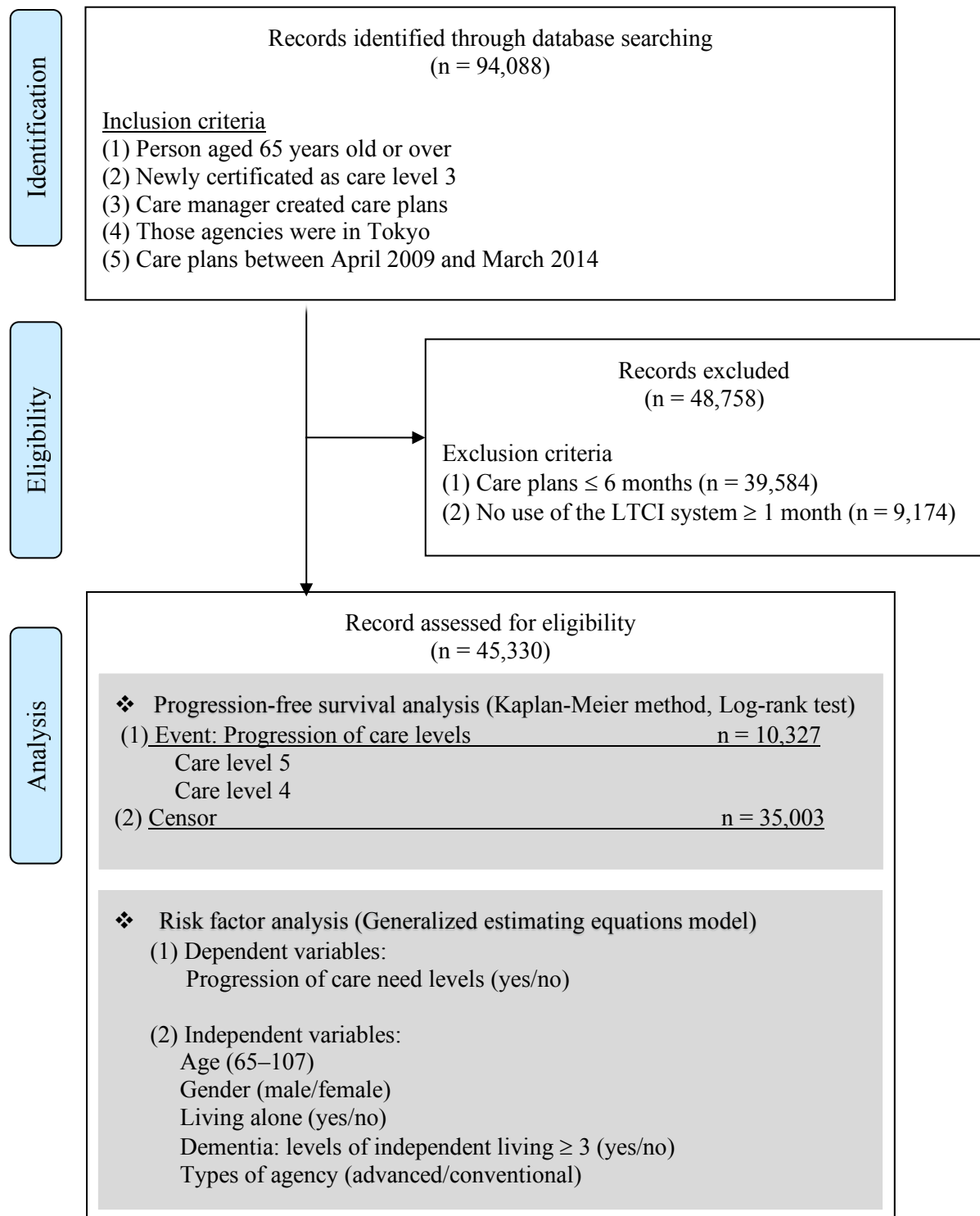

Supplement: Multimedia Appendix 1 [file aging_v1i2e11117_app1.pdf]
