# Supplementary material for: Association Between Advanced Care Management and Progression of Care Needs Level in Long-Term Care Recipients: Retrospective Cohort Study
Source: JMIR Aging. 2018 Jul 25;1(2):e11117. doi: 10.2196/11117 (PMC6716439; doi:10.2196/11117)
Supplement: Multimedia Appendix 2 [file aging_v1i2e11117_app2.pdf]

Supplemental Data 2

Study population treated in long-term care agency in 2009–2014 by type of care management.

		Type of management	
		Advanced care management (n=12903)	Conventional care management (n=32427)
Participant characteristics			
Age (years)		82.8 ± 7.84	82.7 ± 7.82
Gender	Female	8021 (62.2)	20196 (62.3)
	Male	4882 (37.8)	12231 (37.7)
Living alone	Yes	1825 (14.1)	4640 (14.3)
	No	11087 (85.9)	27787 (85.7)
Dementia	Level of independent living ≥3	3457 (27.5)	7805 (24.1)
	No	9356 (72.5)	24622 (75.9)

A total of 29,815,241 data values in 45,330 patients were analyzed during the analysis for five years. The data are presented as number of the patients (percentage) or mean ± standard deviation.
